# The impact and cost of using mobile digital X-ray units to detect pulmonary TB in South Africa

**DOI:** 10.5588/ijtldopen.25.0803

**Published:** 2026-09-15

**Authors:** L. Coetzee, D. Evans, A. Fononda, H. Hausler, L. Booyens, J. Steingo, K. Hirasen, L. Jamieson, M. Kubjane, G. Meyer-Rath

**Affiliations:** 1 Health Economics and Epidemiology Research Office, Faculty of Health Sciences, University of the Witwatersrand, Johannesburg, South Africa;; 2 TB HIV Care, Cape Town, South Africa;; 3 Department of Family Medicine, University of Pretoria, Pretoria, South Africa;; 4 Department of Global Health, School of Public Health, Boston University, Boston, MA, USA.

**Keywords:** digital chest X-ray (dCXR), screening, economics, tuberculosis, cost-effectiveness

## Abstract

**BACKGROUND:**

Digital chest X-ray (dCXR) enables early TB detection where symptoms screening is inadequate. We evaluated the impact and cost of three dCXR delivery models: long-term community-based mobile vans (LT-vans), long-term facility-based containers (LT-containers), and short-term community-based mobile vans (ST-vans), implemented by a non-governmental organisation (NGO) across two South African provinces.

**METHODS:**

Between 2020 and 2022, nine dCXR units were deployed in four high-TB-burden districts for 1–19 months. Screening yield and TB test positivity were collected from electronic databases. Costs were estimated from the provider perspective using micro-costing methods, with both economic and financial costs reported for NGO-led and routine care implementation.

**RESULTS:**

Overall, 34,110 individuals were screened, 8,605 (25.2%) had abnormal dCXR results, and 1,372 TB cases were diagnosed (4.0% positivity). The cost per person screened with dCXR ranged from $39 to $240. LT-vans had the highest TB positivity (10.1%), followed by LT-containers (3.7%) and ST-vans (1.7%). The lowest cost per TB case identified was with LT-vans ($384 economic; $375 financial) under routine care implementation, and the highest with ST-vans ($14,157 economic; $8,741 financial) under NGO-led implementation.

**CONCLUSION:**

Long-term mobile vans were the most cost-effective model, yielding the highest TB rates, while short-term vans offered high volumes but at higher costs.

In 2024, TB was the leading infectious cause of death with 10.7 million new cases and 1.23 million deaths.^1^ South Africa accounts for 3.6% of global TB cases and continues to face major diagnostic gaps, including an estimated 65,000 undiagnosed and untreated cases in 2022.^2,3^ In support of meeting the WHO 2035 target of a 95% reduction in TB deaths from 2010 levels, the National TB Programme Strategic Plan 2023–2028 aims to expand access to high-quality TB screening services to achieve a 95% case detection rate by expanding community-based TB screening and testing using tools that do not depend on symptoms or sputum, to screen priority populations regardless of symptoms and ensure timely linkage to care.^4,5^

Digital chest X-ray (dCXR) has proven feasible and effective in South Africa. A 2022 study, in 23 high-TB-burden South African facilities, reported that standalone dCXR identified 84% of TB cases while using less than half the Xpert MTB/RIF Ultra tests.^6^ Mobile dCXR can also be used to screen individuals from key and vulnerable populations for TB. Mobile dCXR doubled case detection in correctional facilities in South Africa compared with symptom screening alone.^7^ A cross-sectional study in three primary health clinics found that dCXR with computer-aided detection (CAD) yielded a similar proportion of undiagnosed TB cases (1.9%) compared to symptom-based screening (2.0%), but required fewer diagnostic tests.^8^ Community and national surveys in South Africa found that most TB cases, 78% in a rural multi-morbidity survey and 58% in the National TB Prevalence Survey, were asymptomatic and detected only by abnormal chest X-rays.^9,10^

Although mobile dCXR shows promise for early TB diagnosis in South Africa, the optimal implementation model remains unclear,^9^ and evidence comparing the effectiveness and cost-effectiveness of community versus facility-based deployment is limited. To support decision-making, we evaluated the impact, costs, and intermediary cost-effectiveness of three dCXR screening models in two high-TB-burden districts in the Western Cape and KwaZulu-Natal provinces of South Africa.

## METHODS

The National Department of Health, funded by the Global Fund, commissioned TB HIV Care,^11^ a non-governmental organisation (NGO), to provide TB services in high-TB-burden districts from 2019 to 2022 as part of standard-of-care programme activities. Standard-of-care followed the South African national TB guidelines and included testing of symptomatic patients at the primary health care facility, invitations to household contacts for testing, and TB preventative therapy and treatment. Nine dCXR screening units (mobile vans or containers) were deployed across four high-TB-burden districts: the urban Metropolitan Municipalities of eThekwini (KwaZulu-Natal province, KZN), the City of Cape Town (Western Cape province, WC), and the rural districts of King Cetshwayo and Ugu (both KZN). District characteristics are summarised in Supplementary Data Table S1.

The dCXR screening units operated for 1–19 months per district in 2020–2022, targeting high-TB-notification areas and informal settlements, identified through Geographic Information System (GIS) hotspot mapping, using data from the District Health Information System and Three Interlinked Electronic Registers (TIER.Net), and data on population sizes and characteristics and previous TB cases were also used for this purpose (Supplementary Data Table S1). For additional information about the intervention, see Supplementary Data Text S1.

We compared three delivery models for dCXR screening: 1) long-term community-based mobile vans (LT-vans) operating for 16 months in one community, 2) long-term community-based containers (LT-containers) placed next to health care facilities for 5–16 months, and 3) short-term community-based mobile vans (ST-vans) serving the same community for 1 month, introduced during COVID-19 (Table 1). LT-vans used reduced staff and equipment, with moderate consumables and high media outreach. LT-containers were integrated with routine facility workflow, with higher staff and equipment support, increased consumables, and intensified demand creation. ST-vans used substantial staff, minimal equipment, increased consumables, and intensive demand creation and media outreach.

**Table 1.** Placement and operational months of dCXR containers at facilities and mobile vans in communities per district per delivery model.

Model	Long-term vans	Long-term containers	Short-term vans
Short name	LT-vans	LT-containers	ST-vans
Deployment	Community-based	Facility-based	Community-based
Manner	Mobile vans	Containers adjacent to the facility	Mobile vans
Duration of operation	16 months	5–16 months	1 month
Total months in operation across all sites	16 months	62 months	3 months
dCXR screening unit per district (province)	1 in eThekwini (KZN)	2 in eThekwini (KZN) and 3 in City of Cape Town (WC)	1 in City of Cape Town (WC), 1 in King Cetshwayo district (KZN), and 1 in Ugu district (KZN)
Total dCXR screening units	1	5	3
Facility/location	Hotspot areas in one district	Two hospitals, three community health centres, and five primary health care clinics	Hotspot areas in three districts

dCXR = digital chest X-ray; KZN = KwaZulu-Natal; WC = Western Cape.

### Data sources

We obtained de-identified programme data from electronic databases, including screening volumes, dCXR results, laboratory testing, and TB diagnoses (Supplementary Data Table S2 and Table 2). We also collected detailed expenditure data to allocate shared resources, including staff time and consumables, across the dCXR delivery models.

**Table 2.** Parameter definitions.

Parameter	Numerator	Denominator
Average dCXR per month	Total number of dCXRs reported for each model	Number of months dCXR was operational for the model of care
Screening yield of dCXR (%)	Total number of clients with^A^ abnormal dCXR outcomes suggestive of TB	Total number of clients screened by dCXR
Average number of TB cases identified per month	Laboratory or clinically confirmed diagnosis of active TB (TB cases identified)	Number of months dCXR screening unit was operational for the model of care
TB test (dCXR) positivity	Total number of TB cases identified	Total number of clients screened by dCXR
Number needed to screen to identify one TB case	Total number of clients screened by dCXR	Total number of TB cases identified
Average cost per person screened by dCXR	Total estimated cost of each model over the implementation period	Number of dCXR screenings in each dCXR screening unit during the same period
Average cost per client with an abnormal dCXR outcome suggestive of TB	Total cost of each model	Number of abnormal dCXR outcomes suggestive of TB identified for each model
Average dCXR screening cost per TB case identified	Total cost of each model	Number of TB cases identified for each model of care

dCXR = digital chest X-ray.

A An abnormal dCXR suggestive of TB was defined as a chest radiograph classified by computer-aided detection (CAD) as above the pre-specified threshold for presumptive TB, consistent with WHO guidance on the use of CAD for TB screening.^20^

### Cost analysis

Costs were analysed from the provider perspective, using a combination of micro-costing and expenditure analysis. The financial cost was based on actual resource use and expenditure during implementation and included both capital costs, such as equipment, and recurrent costs, including staff, consumables, demand creation, and media coverage. Shared resources were allocated across models based on the proportion of total dCXR screenings performed, further refined by additional quantity measures such as the number of dCXR mobile vans or dCXR containers per district and province to align cost distribution with the operational scale and geographic reach of each model. Capital costs were annualised over the years of useful life as informed by the implementing partners and incorporated into the monthly cost estimates. Economic costs also included estimating the value of donated goods and volunteers’ time, as well as the cost of linkage officers used for mobilisation.

We analysed two scenarios: 1) a baseline scenario representing NGO-led dCXR screening implemented by TB HIV Care within the public health sector and 2) a modelled routine care scenario simulating implementation within the public-sector health system. For the latter, we modified cost inputs, including staff cadres, equipment, supplies, and demand-creation activities, based on expert guidance that reflected typical resource use and quantities (Supplementary Data Table S3), but assumed the same effectiveness as in the NGO-led scenario. We calculated the cost and outcome parameters as defined in Table 2. Monthly costs were estimated by multiplying monthly resources used, unit costs, and patient numbers. Total costs were analysed by delivery model. Average monthly outcomes from the dCXR screening cascade for the three models of care were used to evaluate the cost of four key metrics: 1) TB test (dCXR) positivity, 2) the average cost per person screened using dCXR, 3) the average cost per client with an abnormal dCXR result suggestive of TB, and 4) the average dCXR screening cost per TB case identified. All costs were inflated to 2024 South African Rand (ZAR) using country-specific inflation rates^12^ and converted to 2024 United States dollars (USD) using the 2024 period average of 18.83 ZAR = 1 USD.^13^

### Ethical statement

This analysis relied solely on de-identified data from programme activities and did not involve direct patient contact.

## RESULTS

### Outcomes of dCXR NGO-led implementation

During the 2020–2022 NGO-led implementation as part of standard-of-care programme activities, the three delivery models demonstrated varying performance in monthly screening and TB case detection (Table 3). Community-based LT-vans screened an average of 189 clients per month, with 20 abnormal dCXR results (10.8%) and 19 TB cases identified (10.1%). Facility-based LT-containers screened 434 clients monthly, with 127 abnormal dCXR results (29.3%) and 16 TB cases diagnosed, resulting in a test positivity rate of 3.7%. Community-based ST-vans had the highest throughput, screening 1,366 clients per month, but with a lower proportion of abnormal results (140; 10.2%) and only 23 identified TB cases (1.7%). Across the full NGO-led implementation, 34,110 individuals were screened, resulting in 8,605 abnormal dCXR findings (25.2%) and 1,372 TB cases diagnosed. This corresponds to an overall TB detection rate of 4.0%, with 25 people needing to be screened to identify one TB case.

**Table 3.** dCXR screening cascade for the different models of care, averaged over operational months per model of care.

Model of care	Long-term community-based van	Long-term facility-based container	Short-term community-based van
Total months in operation	16	62	3
	Average per month in operation	Average per month in operation	Average per month in operation
Clients screened by dCXR	189	434	1,366
Clients with normal dCXR outcome	125	381	1,086
Clients with abnormal TB suggestive dCXR outcome	20	127	140
Clients with abnormal dCXR not suggestive of TB	52	65	134
Screening yield of dCXR (%)	10.8%	29.3%	10.2%
TB cases identified	19	16	23
TB test (dCXR) positivity (%)	10.1%	3.7%	1.7%
Number needed to screen to identify one TB case	10	27	59

dCXR = digital chest X-ray.

### Costs

The average economic cost per client screened using dCXR varied across the three models of care, and two scenarios ranged from $39 to $240. The lowest cost observed was in the LT-vans model under routine care, due in part to the lower staff and equipment costs, and the highest cost was in the ST-vans model under the NGO scenario, largely due to demand-creation costs with accounted for 17% of costs (Table 4). The average cost per client with an abnormal dCXR result suggestive of TB and per TB case identified followed a similar pattern, reflecting the differences in the mobile dCXR models’ delivery and scale of implementation.

**Table 4.** Average economic and financial costs of dCXR screening by scenario in 2024 US$.

dCXR unit	Cost per person screened per category (% of total average cost)					Average total cost		
	Staff	Equipment	Consumables	Demand creation	Media	Per person screened by dCXR	Per client with abnormal dCXR outcome suggestive of TB	Per TB case identified
1) Economic cost								
i) NGO scenario								
LT-vans	$139.08	$0.93	$9.57	$13.86	$26.35	$190	$1,758	$1,885
	73%	0.49%	5%	7%	14%			
LT-containers	$164.24	$4.97	$10.77	$27.72	$6.37	$214	$731	$5,761
	77%	2%	5%	13%	3%			
ST-vans	$163.85	$5.55	$10.21	$41.58	$18.49	$240	$2,344	$14,157
	68%	2%	4%	17%	8%			
ii) Routine care scenario								
LT-vans	$21.93	$1.85	$9.58	$5.28	$0	$39	$358	$384
	57%	5%	25%	14%	0%			
LT-containers	$38.19	$4.97	$10.77	$10.55	$0	$64	$220	$1,736
	59%	8%	17%	16%	0%			
ST-vans	$39.49	$5.55	$10.27	$15.83	$0	$71	$696	$4,202
	56%	8%	14%	22%	0%			
2) Financial cost								
i) NGO scenario								
LT-vans	$50.07	$2.97	$9.54	$13.86	$26.35	$103	$952	$1,021
	49%	3%	9%	13%	26%			
LT-containers	$75.23	$4.97	$10.60	$27.72	$6.37	$125	$427	$3,361
	60%	4%	8%	22%	5%			
ST-vans	$74.84	$2.97	$10.10	$41.58	$18.49	$148	$1,447	$8,741
	51%	2%	7%	28%	12%			
ii) Routine care scenario								
LT-vans	$21.93	$0.99	$9.54	$5.28	$0.00	$38	$349	$375
	58%	3%	25%	14%	0%			
LT-containers	$38.19	$4.97	$10.60	$10.55	$0.00	$64	$220	$1,731
	59%	8%	16%	16%	0%			
ST-vans	$39.49	$2.97	$10.17	$15.83	$0.00	$68	$669	$4,043
	58%	4%	15%	23%	0%			

The proportional distribution across cost categories (staff, equipment, etc.) for secondary outcomes is identical to that reported for the average cost per person screened.

dCXR = digital chest X-ray; LT-vans = long-term community-based vans; LT-containers = long-term facility-based containers; ST-vans = short-term community-based vans; NGO = non-governmental organisation–led implementation.

In the NGO scenario, economic costs added 162%–185% to financial costs, primarily due to the inclusion of donated equipment in the LT-vans model and the employment of linkage officers across all models of dCXR screening. In contrast, the routine care scenario resulted in a cost reduction across all three models (20%–52%), primarily driven by leaner staffing structures, minimal equipment use, limited demand-creation activities, and the absence of media engagement.

## DISCUSSION

This study assessed the costs and outcomes of three dCXR delivery models – implemented through either an NGO as part of standard-of-care programme activities or routine implementation within the public-sector health system, across four key metrics: 1) TB test (dCXR) positivity, 2) the average cost per person screened using dCXR, 3) the average cost per client with an abnormal dCXR result suggestive of TB, and 4) the average cost per TB case identified. Findings highlight the value of dCXR, particularly in high-TB-burden hotspot areas – with dCXR units enabling a screening yield of 25% and a TB test (dCXR) positivity rate of 4%, equivalent to one TB case per 25 individuals screened. The yield observed in this study falls just below the 5%–8% range reported for household contact investigations but aligns with the 1.5%–6.7% typically seen in community-based screening among asymptomatic individuals.^14–16^ Results indicate that both dCXR and household contact screening yield one TB case for every 25 individuals screened.^14^ However, mobile dCXR improves resource efficiency by allowing rapid, on-site screening of large numbers of high-risk individuals, reducing repeat visits and personnel time. This efficiency comes with substantial operational costs.

This cost analysis provides early evidence to inform potential integration of dCXR delivery models into routine health care. Evidence on dCXR is emerging, while other TB screening approaches in South Africa, including symptom-based community screening and facility-based active case finding, show varying cost-effectiveness and impact. Costs of dCXR screening vary widely by setting and delivery model. In South Africa, dCXR screening in correctional facilities had an incremental cost-effectiveness ratio of US$22,278 per TB case detected,^17^ an increase of 57% over the economic cost of our least cost-effective model, ST-vans in the NGO scenario. In Malawi, dCXR with CAD costs US$19.92 per participant screened, much lower than our range of $39–$240 per client screened across models of care and scenarios.^18^ Similar to our results, mobile screening in India revealed high capital and operational costs, with staffing and equipment as major cost drivers.^19^ When considering the costs and impacts of scaling up national TB programme interventions in South Africa, community-based dCXR interventions show modest impact on TB incidence and mortality and are less cost-effective than alternative TB screening strategies.^20^ Although dCXR shows promise in underserved areas, differences in costing approaches and short follow-up periods limit generalisability, underscoring the need for standardised, long-term evaluations.^21^

We found variation in the outcomes and costs of dCXR screening across three delivery models and two implementation scenarios. ST-vans yielded threefold higher monthly screening volumes than LT-containers, possibly due to the higher demand creation and media coverage. LT-containers yielded a higher percentage of abnormal dCXR suggestive of TB than LT- and ST-vans, likely reflecting integration into the health facility’s workflows and routine screening. LT-containers yielded higher TB positivity than ST-vans, possibly due to at-risk and vulnerable groups like PLHIV seeking or accessing treatment at the health care facility. The cost per person screened and cost per TB case found were lowest in LT-vans. This likely reflects smaller staff contingents and lower demand-creation costs in comparison to the ST-vans.

The LT-vans model consistently had the lowest cost per person screened and per TB case identified, particularly under routine care. In contrast, the ST-vans model showed the highest average costs, with demand creation and media accounting for 22%–41% of recurrent costs. The LT-containers model fell between the two, with moderate use of outreach strategies. Across all models, NGO implementation was more expensive than routine care, due to the broader staffing and resource requirements.

Staff salaries formed the largest cost component in all models, while cost differences were driven by the intensity of media use and demand-creation activities. The ST-vans model, implemented as a short-term, high-intensity intervention, relied heavily on these components to boost reach, increasing costs. In contrast, the LT-vans minimised costs through streamlined staffing and donated equipment in the NGO setting. Cost-effective TB screening depends on aligning programme intensity to resource availability and the intended scale. For routine implementation, low-intensity community-based approaches may be more sustainable, with targeted demand creation used selectively.

Implementers and policymakers should consider these cost drivers when scaling up dCXR-based TB screening. Cost-effectiveness can be improved by adopting lower-cost models, such as the LT-vans, and managing outreach strategies to reduce unnecessary expenditures. Future research should explore balancing community engagement with operational efficiency and assess the long-term sustainability of different dCXR models in public health care settings.

Both the NGO-led implementation and our cost analysis have limitations. First, only one LT-van was implemented due to COVID-19-related delays, limiting assessment of cost variability and generalisability. Second, short-term ST-van deployment due to COVID-19 delays may have inflated average costs. With broader rollout and operational efficiencies, the average cost per person screened and per TB case identified are likely to decrease. Third, the delivery models focused on high-burden settings and informal settlements identified through geospatial hotspot mapping, so findings may not be generalisable to lower-burden parts of South Africa, where the performance, feasibility, and cost-effectiveness of dCXR may differ and would require further investigation. Fourth, the routine care scenario was based on assumptions about typical public-sector resources, which may underestimate resources needed to sustain effective community engagement over time; since we did not additionally change effectiveness in this scenario, we might have under- or overestimated reach and yield of this hypothetical scenario. In practice, communities often show strong initial interest in novel services; however, engagement may decline without continued outreach and demand creation.

The feasibility of implementing dCXR screening under routine care remains uncertain. While cost assumptions were applied consistently for comparability, routine care implementation may require additional investments, potentially affecting the cost-effectiveness of these models. Further research is needed to assess the long-term sustainability and scalability of dCXR interventions at health care facilities and in communities under routine health system conditions.

## CONCLUSION

Long- and short-term community-based mobile van dCXR screening within communities identified by GIS hotspot mapping reaches high-risk clients in underserved areas who do not typically present at health facilities. Long-term container dCXR screening at health facilities adds asymptomatic clients typically missed by TB symptom screening. Short-term mobile vans in communities increased monthly screening volumes, while long-term containers at health care facilities yielded a higher percentage of abnormal dCXR suggestive of TB. Facility-based containers yielded higher screening positivity than ST-vans. Costs per person screened and per TB case found were lowest in LT-vans, comparing favourably with other TB screening interventions and offering an alternative to existing screening modalities if budgets allow. Due to high unit costs and coverage limitations, dCXR rollout should be limited to high-burden districts with overburdened facilities, maximising its impact alongside existing TB services.

## References

[1] World Health Organization. Global Tuberculosis Report 2025. Geneva: WHO, 2025.

[2] Chakaya J, Global Tuberculosis Report 2020–reflections on the global TB burden, treatment and prevention efforts. Int J Infect Dis. 2021;113(Suppl 1):S7-S12. 33716195 10.1016/j.ijid.2021.02.107 PMC8433257

[3] TB Accountability Consortium. The state of TB in South Africa: from crisis to opportunity: accelerating testing to end TB. Johannesburg, South Africa: TB Accountability Consortium, 2025.

[4] World Health Organization. Gear up to end TB: introducing the end TB strategy. Geneva: WHO, 2015.

[5] South Africa Government, National Department of Health. National strategic plan for HIV TB and STIs 2023-2028. Cape Town, South Africa: National Department of Health, 2023.

[6] Connell L, Dlamini T, Okonji E. Using dCXR with CAD to augment TB screening in primary healthcare facilities in South Africa. Johannesburg, South Africa: Right to Care, 2003.

[7] Velen K, Digital chest x-ray with computer-aided detection for tuberculosis screening within correctional facilities. Ann Am Thorac Soc. 2022;19(8):1313-1319. 34914539 10.1513/AnnalsATS.202103-380OC

[8] Moodley N, Digital chest radiography enhances screening efficiency for pulmonary tuberculosis in primary health clinics in South Africa. Clin Infect Dis. 2022;74(9):1650-1658. 34313729 10.1093/cid/ciab644

[9] Fehr J, Computer-aided interpretation of chest radiography reveals the spectrum of tuberculosis in rural South Africa. NPJ Digit Med. 2021;4(1):106. 34215836 10.1038/s41746-021-00471-y PMC8253848

[10] South Africa National Department of Health. The First National TB Prevalence Survey: South Africa 2018. Pretoria, South Africa: The Department, 2021.

[11] TB HIV Care. https://tbhivcare.org/.

[12] International Monetary Fund. Inflation rate, average consumer prices, 2022.

[13] OFX. Historical exchange rates. London, UK: UKForex Limited, 2025. http://www.usforex.com/forex-tools/historical-rate-tools/yearly-average-rates.

[14] Little KM, Yield of household contact tracing for tuberculosis in rural South Africa. BMC Infect Dis. 2018;18(1):299. 29973140 10.1186/s12879-018-3193-7 PMC6030742

[15] Kranzer K, Feasibility, yield, and cost of active tuberculosis case finding linked to a mobile HIV service in Cape Town, South Africa: a cross-sectional study. PLoS Med. 2012;9(8):e1001281. 22879816 10.1371/journal.pmed.1001281 PMC3413719

[16] Berhanu RH, Yield of facility-based targeted universal testing for tuberculosis with Xpert and mycobacterial culture in high-risk groups attending primary care facilities in South Africa. Clin Infect Dis. 2023;76(9):1594-1603. 36610730 10.1093/cid/ciac965 PMC10156124

[17] Kim H, Symptom and digital chest X-ray TB screening in South African prisons: yield and cost-effectiveness. Int J Tuberc Lung Dis. 2020;24(3):295-302. 32228759 10.5588/ijtld.19.0214

[18] MacPherson P, Computer-aided X-ray screening for tuberculosis and HIV testing among adults with cough in Malawi (the PROSPECT study): a randomised trial and cost-effectiveness analysis. PLoS Med. 2021;18(9):e1003752. 34499665 10.1371/journal.pmed.1003752 PMC8459969

[19] Datta B, Comparison of clinical and cost-effectiveness of two strategies using mobile digital x-ray to detect pulmonary tuberculosis in rural India. BMC Public Health. 2019;19:1-8. 30669990 10.1186/s12889-019-6421-1 PMC6341675

[20] Kubjane M, The cost and impact of scaling up South African National Tuberculosis Programme interventions: a cost-effectiveness analysis. Abstracts of the 54th Union World Conference on Lung Health, Paris, France, 15-18 November 2023.

[21] World Health Organization. WHO consolidated guidelines on tuberculosis. Module 2: screening – systematic screening for tuberculosis disease. Geneva: WHO, 2021. 33822560

